# A survey of perceived training differences between ophthalmology residents in Hong Kong and China

**DOI:** 10.1186/s12909-015-0440-0

**Published:** 2015-09-28

**Authors:** Alvin L. Young, Vishal Jhanji, Yuanbo Liang, Nathan Congdon, Simon Chow, Fenghua Wang, Xiujuan Zhang, Xiaofei Man, Mingming Yang, Zhong Lin, Hunter GL Yuen, Dennis SC Lam

**Affiliations:** 1 Department of Ophthalmology and Visual Sciences, Prince of Wales Hospital, Hong Kong SAR, Hong Kong; 2 Department of Ophthalmology and Visual Sciences, The Chinese University of Hong Kong, Hong Kong SAR, Hong Kong; 3 The Affiliated Eye Hospital, School of Optometry and Ophthalmology, Wenzhou Medical University, No. 270 West College Road, Wenzhou, Zhejiang 325027 China; 4 Centre for Eye Research Australia, University of Melbourne, Melbourne, VIC Australia; 5 Zhongshan Ophthamic Center, Sun Yat-sen University, Guangzhou, China; 6 Orbis International, New York, USA; 7 Queen’s University Belfast, Belfast, UK; 8 Beijing Tongren Eye Center, Beijing Tongren Hospital, Capital Medical University; Beijing Ophthalmology & Visual Science Key Lab, Beijing, China; 9 Hong Kong Eye Hospital, Hong Kong SAR, Hong Kong

**Keywords:** Residency, Training, Ophthalmology, China, Hong Kong, Cataract

## Abstract

**Background:**

To study the differences in ophthalmology resident training between China and the Hong Kong Special Administrative Region (HKSAR).

**Methods:**

Training programs were selected from among the largest and best-known teaching hospitals. Ophthalmology residents were sent an anonymous 48-item questionnaire by mail. Work satisfaction, time allocation between training activities and volume of surgery performed were determined.

**Results:**

50/75 residents (66.7 %) from China and 20/26 (76.9 %) from HKSAR completed the survey. Age (28.9 ± 2.5 vs. 30.2 ± 2.9 years, *p* = 0.15) and number of years in training (3.4 ± 1.6 vs. 2.8 ± 1.5, *p* = 0.19) were comparable between groups. The number of cataract procedures performed by HKSAR trainees (extra-capsular, median 80.0, quartile range: 30.0, 100.0; phacoemulsification, median: 20.0, quartile range: 0.0, 100.0) exceeded that for Chinese residents (extra-capsular: median = 0, *p* < 0.0001; phacoemulsification: median = 0, *p* < 0.0001). Chinese trainees spent more time completing medical charts (>50 % of time on charts: 62.5 % versus 5.3 %, *p* < 0.0001) and received less supervision (≥90 % of training supervised: 4.4 % versus 65 %, *p* < 0.0001). Chinese residents were more likely to feel underpaid (96.0 % vs. 31.6 %, *p* < 0.0001) and hoped their children would not practice medicine (69.4 % vs. 5.0 %, *p* = 0.0001) compared HKSAR residents.

**Conclusions:**

In this study, ophthalmology residents in China report strikingly less surgical experience and supervision, and lower satisfaction than HKSAR residents. The HKSAR model of hands-on resident training might be useful in improving the low cataract surgical rate in China.

## Background

Some 90 % of preventable blindness occurs in the developing world [1], and with the aging of populations globally, there is a rapidly increasing demand for well-trained ophthalmic personnel. Data from the International Council of Ophthalmology (ICO) reveal a shortage of ophthalmologists, with a shortfall predicted in both low income and developed areas [2].

China accounts for 18 % of the world’s blind, with an estimated 5 million people who have lost the ability to self-care, half of them due to un-operated cataract [3, 4]. The Chinese Ministry of Health has made it a priority to tackle preventable blindness nationally, but the estimated number of cataract operations performed annually (360,000) falls short of the number becoming blind each year from cataract (400,000) [3]. The cataract surgical rate (CSR) is the number of cataract operations per million population per year. China’s cataract surgical rate of 900 is far lower than that of neighboring India (5600) [5] and Vietnam (1900) [6], both of whom have lower per capita incomes [7].

In addition to the lack of access to affordable rural eye care [8, 9] and the aging Chinese population [10], an important cause of China’s cataract problem is the lack of qualified ophthalmologists. Fewer than 36 % of the estimated 28,000 ophthalmologists in China perform cataract surgery [11], and quality among those who do operate remains problematic, especially in rural areas [12–14]. There is nothing in the evidence presented to justify “poorer outcomes”, lower output is true.

Structured, hands-on residency training is critical to produce a national cadre of capable surgeons [15, 16]. Lack of optimal training could potentially contribute to lower output and poorer outcomes. The current report examines for the first time the attitudes, time allocation and self-reported surgical experience of ophthalmology residents in China and the Hong Kong Special Administrative Region (HKSAR), whose medical and training systems remain separate from those in the People’s Republic [17].

## Methods

The data were collected via an anonymous questionnaire from Chinese and HKSAR ophthalmology residents between January 2012 and March 2012. The ethics committee of the Chinese University of Hong Kong approved the protocol for this project. The study followed the tenets of the Declaration of Helsinki.

### Subjects and institutions

University-based and government hospitals were selected from the HKSAR and China. Five purposeful hospitals in China were identified based on reputation and size of the training program, national university rankings [18] and geographic diversity, with programs from the north, south, east and west of the country. The largest institutions (*n* = 2) were selected in the HKSAR, based both on clinical volume and the number of ophthalmology residents in the program. The Chinese hospitals included in this study performed 20,000 to 50,000 ophthalmic surgeries annually, with annual outpatient visits ranging from 400,000 to 1,000,000. Each hospital had 20-70 senior consultant ophthalmologists. The number of outpatient visits at HKSAR hospitals ranged from approximately 150,000 to 200,000 annually, with the annual surgical volume at each approaching 6000. Each of the HKSAR facilities had approximately 10 consultant ophthalmologists.

### Questionnaire

A questionnaire consisting of 48 questions in Chinese was designed to obtain information from resident trainees on: 1) Working environment and satisfaction; 2) Clinical exposure, supervision and hands-on training opportunities; 3) Involvement in academic and research activities. Demographic information including gender, age, educational level and years of training was also recorded. Questionnaires were sent by standard mail to all resident ophthalmologists at the selected programs, with a covering letter clearly explained the purpose of the questionnaire. We contacted the appropriate person in the administrative office at each institution to ensure that all ophthalmology residents received the questionnaire forms. The form was pilot-tested for clarity and usability on Chinese graduate students studying in the HKSAR, and revised according to their feedback. All respondents in the actual study were ophthalmology residents who had not yet completed their specialist instruction.

The questionnaire was self-administered anonymously, requiring approximately 45 min to complete. Respondents were instructed not to discuss the questionnaires amongst themselves at any stage. Data forms were returned by post to the coordinating center at the Chinese University of Hong Kong, where the data were extracted and analyzed. It was explained to potential participants that returning the form was indicative of their providing formed consent, so that anonymity could be maintained without need for a signature.

### Statistical methods

Responses to the various questions were summarized as percentages or mean/median values. Student’s *t*-test or Fisher’s exact test (in the case of small values in cells) were used to compare the distribution of responses between subjects in China and the HKSAR. Bonferroni correction was utilized when interpreting the results of multiple comparisons in this study. There were 21 comparisons, and thus the Bonferroni-adjusted alpha was 0.05/21 = 0.0024. The Statistical Analysis System for Windows version 9.1.3 (SAS Inc., Cary, NC) was used for all analyses.

## Results

### Participant characteristics

Twenty (17 females, 85 %) ophthalmology residents with a response rate of 76.9 % (20/26) were recruited from the HKSAR and 50 (34 females, 68 %, *p* = 0.23) with a response rate of 66.7 % (50/75) from China. Mean age (China 30.2 ± 2.9;HKSAR: 28.9 ± 2.5 years, *p* = 0.15) and duration of training (China 2.8 ± 1.5 years; HKSAR 3.4 ± 1.6 years, *p* = 0.19) at the time of enrollment did not differ significantly between the two groups. A higher proportion of Chinese trainees reported having a masters or doctoral degree than for the HKSAR (80.0 % vs. 15.0 %, *p* < 0.0001) (Table 1).

**Table 1:** Demographic characteristics of participating residents in China and Hong Kong

	China	Hong Kong	*P* value
Number	50	20	
Gender (male: female)	16: 34	3:17	0.23^*^
Age (years, mean ± SD)	30.1 ± 2.9	28.9 ± 2.5	0.15^**^
Years of training (years, mean ± SD)	2.8 ± 1.5	3.4 ± 1.6	0.19^*^
Years of training (N, %)			
Year 1	14 (28.0)	3 (15.0)	0.30^*^
Year 2	9 (18.0)	3 (15.0)	
Year 3	8 (16.0)	6 (30.0)	
Year 4	11 (22.0)	2 (10.0)	
Year 5	7 (14.0)	4 (20.0)	
Year 6	1 (2.0)	2 (10.0)	
Highest educational degree (N, %)			
Bachelor	10 (20)	17 (85.0)	<0.0001^*^
Masters or PhD (No., %)	40 (80.0)	3 (15.0)	
Hospital			
Hong Kong 1		9	
Hong Kong 2		11	
China 1	6		
China 2	10		
China 3	13		
China 4	12		
China 5	9		

*SD* Standard deviation(50/58)

^*^Fisher’s exact test

^**^Student’s *t*-test

### Work environment and satisfaction

Work hours were longer for respondents felt from China than for those from the HKSAR (worked ≥ 60 h per week: 60.0 % vs. 33.3 %, *P* = 0.052), though the difference was not significant with Bonferroni correction. Chinese trainees spent significantly more time completing medical charts than did those from HKSAR (proportion spending > 50 % of their time on charts: 62.5 % versus 5.3 %, *p* < 0.0001, Table 2). The majority of the Chinese trainees indicated that they felt they were underpaid (96.0 %) and did not want their children to practice medicine (69.4 %). Only 31.6 % (*P* < 0.0001) and 5 % (*P* < 0.0001) of the HKSAR trainees respectively shared similar sentiments (Table 2).

**Table 2:** Distribution of interviewee responses: Working environment

	China	Hong Kong	*P* value^*^
Working time (hours per week, including both clinical and research activities)			
40	2 (4.0)	0 (0.0)	0.20
45	2 (4.0)	2 (11.1)	
50	7 (14.0)	4 (22.2)	
55	9 (18.0)	6 (33.3)	
60	8 (16.0)	4 (22.2)	
65	10 (20.0)	1 (5.6)	
70	12 (24.0)	1 (5.6)	
Proportion of time spent in completing medical documents (such as patient charts)			
≤10 %	2 (4.2)	8 (42.1)	<0.0001
≤20 %	2 (4.2)	5 (26.3)	
≤30 %	9 (18.8)	3 (15.8)	
≤40 %	5 (10.4)	2 (10.5)	
≤50 %	8 (16.7)	1 (5.3)	
≤60 %	10 (20.8)	0 (0.0)	
>60 %	12 (25.0)	0 (0.0)	
Salary satisfaction			
Earned > deserved	0 (0.0)	0 (0.0)	<0.0001
Earned = deserved	2 (4.0)	13 (68.4)	
Earned < deserved	48 (96.0)	6 (31.6)	
Do you want your children to practice medicine?			
Yes	1 (2.0)	8 (40.0)	<0.0001
No	34 (69.4)	1 (5.0)	
Respect child’s choice	14 (28.6)	11 (55.0)	
Missing	1 (2.0)		

^*^Fisher’s exact test

### Clinical experience

While 26.5 % (13/49) and 10.4 % (5/49) of the PRC trainees felt that they could not independently use 90D and 20D lenses respectively, 100 % (20/20) of HKSAR trainees felt confident in using these lenses on a daily basis (*p* = 0.31 and *p* = 0.014 respectively) after completing one year of training. The number of retinal barrier laser treatments for retinal holes (median: 50.0, quartile range: 3.0, 150.0) and YAG posterior capsulotomies (median 50.0, quartile range: 3.0, 100.0) performed by HKSAR trainees significantly exceeded the figures reported among Chinese trainees (barrier laser: median = 0, *P* < 0.0001; capsulotomies: median = 0, *P* < 0.0001) (Table 3). The number of cataract procedures reported by HKSAR trainees (extra-capsular, median 80.0, quartile range: 30.0, 100.0; phacoemulsification, median: 20.0, quartile range: 0.0, 100.0) was also greater than that for Chinese residents (extra-capsular: median = 0, *P* < 0.0001; phacoemulsification: median = 0, *P* < 0.0001) (Table 3). Limiting the comparison to senior residents highlights the difference in training experience even further: 8 Hong Kong residents with > 3 years of experience completed a mean of 125 ECCE and 100 Phaco procedures, compared with means of 0 and 0 respectively for 19 Chinese residents at the equivalent level of training (Table 4).

**Table 3:** Distribution of interviewee responses: Surgical output

	China	Hong Kong	*P* value^*^
Laser treatments (median, upper and lower quartiles)			
Laser treatment of retinal hole	0.0 (0.0, 0.0)	50.0 (3.0, 150.0)	<0.0001
Cyclophotocoagulation	0.0 (0.0, 0.5)	10.0 (1.0, 20.0)	<0.0001
YAG Iridectomy	0.0 (0.0, 0.0)	40.0 (5.0, 100.0)	<0.0001
YAG Posterior capsulotomy	0.0 (0.0, 0.0)	50.0 (3.0, 100.0)	<0.0001
Surgery (> = 80 % of the entire procedure performed by resident) (median, upper and lower quartiles)			
Chalazion	12.0 (0.0, 50.0)	0.0 (0.0, 30.0)	0.21
Pterygium	9.0 (0.0, 33.0)	20.0 (12.5, 50.0)	0.04
Entropion	2.0 (0.0, 5.5)	3.0 (0.0, 10.0)	0.72
ECCE + IOL	0.0 (0.0, 0.0)	80.0 (30.0, 100.0)	<0.0001
Phacoemulsification + IOL	0.0 (0.0, 0.0)	20.0 (0.0, 100.0)	<0.0001

*ECCE* Extracapsular cataract extraction; *IOL* Intraocular lens; *YAG* Yttrium aluminium garnet

^*^Wilcoxon test

**Table 4:** Number (median, lower and upper quartiles) of ECCE and phaco operations performed by residents in mainland China and Hong Kong

	ECCE		Phaco	
	ML	HK	ML	HK
Junior residents	0 (0, 0)	30 (2, 100)	0 (0, 0)	0 (0, 20)
Senior residents	0 (0, 2)	125 (80, 200)	0 (0, 0)	100 (90, 250)

Junior residents were defined as residents of the first year to the third year, while senior residents defined as residents of the fourth year to the sixth year

Supervision by senior doctors was also less available for trainees from China: 65 % of HKSAR trainees reported that ≥ 90 % of their training occurred with adequate supervision, while only 4.4 % of Chinese trainees reported this level of supervision (*P* < 0.0001), and 62.6 % of PRC trainees reported that ≤30 % of their training occurred under supervision (Table 5).

**Table 5:** Distribution of interviewee responses: Supervision and independent interpretation of examinations

	Hong Kong	Mainland	*P* value^*^
Percentage of surgeries supervised by senior doctors			
≤ 10 %	1 (5.0)	11 (24.4)	<0.0001
≤ 30 %	1 (5.0)	17 (37.8)	
≤ 50 %	2 (10.0)	12 (26.7)	
≤ 70 %	3 (15.0)	3 (6.7)	
90 %	6 (30.0)	2 (4.4)	
100 %	7 (35.0)	0 (0.0)	
Missing		5 (10.0)	
Examinations capable of interpreting independently			
Visual field	18 (90.0)	40 (80.0)	0.49
Fundus fluorescein angiography	17 (85.0)	24 (48.0)	0.007
B-scan ultrasound	19 (95.0)	43 (86.0)	0.42
Optical Coherence Tomography	18 (90.0)	43 (86.0)	1.00
Ultrasound biomicroscopy	10 (50.0)	40 (80.0)	0.02
Heidelberg Retina Tomography	12 (60.0)	23 (46.0)	0.43

^*^Fisher’s exact test

Self-reported ability to interpret examination results independently was comparable between subjects from China, and the HKSAR, with > 50 % of both groups able to interpret visual field testing, ultrasonography and optical coherence tomography independently. Trainees from the HKSAR were more confident in interpreting fluorescein angiograms (85 % vs. 48 %, *p* = 0.007) while their PRC counterparts reported greater familiarity with ultrasound biomicroscopy (80 % vs. 50 %, *p* = 0.02), though the latter difference was not significant with Bonferroni correction (Table 5).

### Research experience

Chinese trainees appeared to place more emphasis on research and academic activities, though differences were not significant after Bonferroni adjustment: 64 % of Chinese trainees reported allocating ≥ 20 % of their time to research, while 35 % of the HKSAR group reported doing so (*P* = 0.03). The number of reported publications per year of training for Chinese trainees was 1.39 ± 2.43 and 0.78 ± 1.32 in Chinese and English-language journals respectively, while HKSAR residents reported 0.57 ± 0.30 publications/year, all in English journals (*P* = 0.37). The major stated purpose of research was to attain promotion (32.7 %) for the PRC trainees, while interest in research (38.2 %) was the predominant reason cited by the HKSAR residents (*P* = 0.038) (Table 6).

**Table 6:** Distribution of interviewee responses: Research

	Hong Kong	Mainland	*P* value
Proportion of total working time spent on research			
None	1 (5.0)	7 (14.0)	0.01^*^
10 %	12 (60.0)	11 (22.0)	
20 %	7 (35.0)	20 (40.0)	
50 %	0 (0.0)	10 (20.0)	
> 70 %	0 (0.0)	2 (4.0)	
Number of publications per year in training			
Chinese	0	1.39	
English	0.57	0.78	0.37^**^
Purpose of doing research			
Interest	13 (38.2)	23 (20.9)	0.03^*^
Promotion	3 (8.8)	36 (32.7)	
Solving clinical problems	10 (29.4)	21 (19.1)	
Improving academic status	4 (11.8)	13 (11.8)	
Improving clinical skills	4 (11.8)	17 (15.5)	

^*^Fisher’s exact test

^**^Student’s *t*-test

## Discussion

Our results indicate that ophthalmology residents perform significantly less surgery during training in China than in the HKSAR. This appears to reflect in part different training standards between the two regions: the current minimum standard for cataract surgery is 100 cases by the completion of residency training in the HKSAR [19] while the Chinese Ophthalmological Society has proposed a target of 15 cases [20], although our data suggest that residents may be falling far short of this figure. By comparison, the United States [21] and Singapore [22] demand 86 independent cataract surgical cases by the completion of training and the United Kingdom requires 350 [23].

A recent report indicates that nearly 90 % of ophthalmology residents in the United States have served as the primary surgeon in more than 100 cataract cases by the completion of residency [24]. Based on a retrospective chart review of 375 surgical records from 25 ophthalmology residents, Wiggins [25] has suggested that residents may need to perform at least 121 cataract cases, as their phacoemulsification time only reached that of senior doctors at this point.

We also found that direct supervision from senior physicians was much less common in Chinese residency training when compared to the HKSAR. Supervised hands-on training is critical to the learning process. The existing literature generally supports the value of mentorship in training residents in ophthalmology [26] and other disciplines [27], but provides very little data on the proportion of resident training occurring under direct supervision in other settings. Thomas et al have documented similar shortcomings in mentoring and comprehensive training of ophthalmology residents in India [28].

Our results suggest that ophthalmic trainees in the PRC are not receiving adequate clinical training compared to international recommendations in part because of the large amount of time spent on paperwork, and to some extent research. The strong emphasis on paperwork [29] and research [30] in the Chinese system of medical training has been reported elsewhere. Recent publications [30] have questioned the value of mandatory research requirements during residency training in western settings [31, 32].

It may be that the limited opportunities for clinical training, perception of being underpaid and high burden of paperwork are causally linked to the dis-satisfaction expressed by Chinese ophthalmology residents, as in their desire not to have their children follow their career path. The annual salary of ophthalmology residents in HKSAR ranges from US$90,400 to 123,500 from first to sixth year of training, while in China the corresponding annual range is US$8400 to 14,400. The 2014 per capita gross domestic product (GDP) as reported by the International Monetary Fund (http://en.wikipedia.org/wiki/List_of_countries_by_GDP_(PPP)_per_capita) for HKSAR was $54,772 and for China $12,880. Thus, the salary for a first year resident in HKSAR was 165 % of GDP and that in the PRC was 65 % of GDP. This low level of satisfaction has important implications for attracting talented young people to the field of ophthalmology in China.

These findings about the state of residency training in China are relevant to the major shortage of surgical output currently facing the country. The CSR in China is currently < 1000 cases/million/year, far behind the volume in poorer neighboring countries such as India (5500) [5] and Vietnam (1900) [6]. By contrast, the CSR in HKSAR is > 5000 [33]. In its current 5-year Blindness Prevention Plan [34], the Chinese Ministry of Health has identified lack of trained ophthalmic personnel as a major stumbling block to improving access to high-quality eye care. Our results suggest that reforms in the resident education system are needed to accomplish this goal, as the Chinese ophthalmology residents surveyed in the current report appear unprepared to manage cataract, the leading cause of blindness in China [35], at their current level of training.

How to resolve this problem? In the United States, residency training programs which do not provide adequate hands-on training for residents are at risk of losing their accreditation from the Accreditation Council for Graduate Medical Education or similar bodies [36]. In HKSAR, the College of Ophthalmology of Hong Kong also conducts periodic review inspection of centers, and substandard training centers may lose the right to train residents [17]. No such consequences exist for training bodies in China.

While ophthalmic training in both HKSAR and China lasts for six years in general, and residents in both settings tend to receive all of their residency training at a single institution, there are important differences. Ophthalmology residents in the HKSAR are trained in public hospitals under the jurisdiction of a body known as the Hospital Authority, with training content regulated according to the guidelines of the Hong Kong College of Ophthalmology. Unlike the situation in HKSAR and many other settings, where a single pathway is available for specialty clinical training in ophthalmology, in China multiple venues provide options for young doctors seeking their initial hands-on clinical experiences. In addition to formal residency training, where hands-on opportunities may be limited, graduate training at the masters and doctoral level is often expected to include clinical rotations. Finally, surgical programs in rural areas offered by non-governmental organizations such as Lifeline Express are also an increasingly popular venue for the training of young doctors, though these experiences may or may not be supervised. Few criteria exist at the national level for the quantity and quality of training offered through these adjunct pathways. A system of standards and enforcement paralleling that in the HKSAR and many other countries appears to be needed for resident training in China. Published recommendations by the International Council of Ophthalmology (ICO) [37] and the Asia-Pacific Academy of Ophthalmology (APAO) [38] provide helpful guidelines for establishing such standards.

## Conclusions

The strengths of the current report are that it provides for the first time an overview of important differences between ophthalmology resident training in China and the nearby HKSAR. The same questionnaires and protocol were used to gather data from two systems under the same government, bringing to light an important problem in view of China’s low CSR. Weaknesses include the small numbers of both programs and residents and lack of randomized selection of programs, both resulting in some uncertainty with regard to the representativeness of the sample. To some extent the resulting bias is deliberate: we preferentially-selected only the best-known training centers in China in order to avoid the potential criticism that the problem of lack of hands-on training is associated mostly or in part with second-tier institutions. If this problem is widespread at the best known institutions, it seems a reasonable inference that less-respected training centers may be equally affected, though we cannot be certain of this. It must also be acknowledged the numbers of surgeries and other data were self-reported and thus also subject to bias, though deliberate under-reporting of surgical experience does not seem likely.

However, given the very large and consistent reported disparities, it seems likely that these results represent real differences in residency training between China and the more typical international instruction system used in the HKSAR. Further studies are warranted to document resident training practices in China, and most importantly, to determine what interventions might be successful in strengthening these systems. Data from other settings would allow our primary hypothesis to be tested directly, namely that fewer hands-on training opportunities during residency training are correlated with lower rates of later surgical service output, particularly for cataract, the world’s leading cause of blindness.
